# A nomogram model for the risk prediction of type 2 diabetes in healthy eastern China residents: a 14-year retrospective cohort study from 15,166 participants

**DOI:** 10.1007/s13167-022-00295-0

**Published:** 2022-08-16

**Authors:** Tiancheng Xu, Decai Yu, Weihong Zhou, Lei Yu

**Affiliations:** 1 Department of Hepatobiliary Surgery, Nanjing Drum Tower Hospital, The Affiliated Hospital of Nanjing University Medical School, No. 321 Zhongshan Road, Nanjing, China; 2 Department of Health Management Centre, Nanjing Drum Tower Hospital, The Affiliated Hospital of Nanjing University Medical School, No. 321 Zhongshan Road, Nanjing, China

**Keywords:** Type 2 diabetes, Nomogram, Risk factor, Predictive preventive personalized medicine

## Abstract

**Background:**

Risk prediction models can help identify individuals at high risk for type 2 diabetes. However, no such model has been applied to clinical practice in eastern China.

**Aims:**

This study aims to develop a simple model based on physical examination data that can identify high-risk groups for type 2 diabetes in eastern China for predictive, preventive, and personalized medicine.

**Methods:**

A 14-year retrospective cohort study of 15,166 nondiabetic patients (12–94 years; 37% females) undergoing annual physical examinations was conducted. Multivariate logistic regression and least absolute shrinkage and selection operator (LASSO) models were constructed for univariate analysis, factor selection, and predictive model building. Calibration curves and receiver operating characteristic (ROC) curves were used to assess the calibration and prediction accuracy of the nomogram, and decision curve analysis (DCA) was used to assess its clinical validity.

**Results:**

The 14-year incidence of type 2 diabetes in this study was 4.1%. This study developed a nomogram that predicts the risk of type 2 diabetes. The calibration curve shows that the nomogram has good calibration ability, and in internal validation, the area under ROC curve (AUC) showed statistical accuracy (AUC = 0.865). Finally, DCA supports the clinical predictive value of this nomogram.

**Conclusion:**

This nomogram can serve as a simple, economical, and widely scalable tool to predict individualized risk of type 2 diabetes in eastern China. Successful identification and intervention of high-risk individuals at an early stage can help to provide more effective treatment strategies from the perspectives of predictive, preventive, and personalized medicine.

## Introduction

Diabetes is a common chronic disease, with considerably high rates of morbidity and mortality [1], and researchers estimate that there were 451 million diabetic patients (5.5% of the global population) worldwide in 2017 and that these numbers will increase to 693 (10.9%) million by 2045 [2]. In addition, an estimated 374 million people suffer from impaired glucose tolerance (IGT) [3]. According to the World Health Organization (WHO), in 2019, an estimated 1.5 million people died from diabetes directly and 2.2 million people died from hyperglycemia in 2012. More than 90% of diabetes is type 2 diabetes (T2DM) [4, 5], and its clinical symptoms are usually not so outwardly apparent. Therefore, the disease may not be diagnosed until several years after its onset, after complications have already appeared. Hence, diagnostic delay is a critical factor that contributes to overall disease controllability and risk for complications [6]. The white paper of the ‘European Association for Predictive, Preventive and Personalized Medicine (PPPM)’ (EPMA) mentions that a key step in the prevention of T2DM is the identification of high-risk individuals [7]. Although genetic structure may partially determine an individual’s response to environmental changes, the main drivers of the global type 2 diabetes epidemic are increases in obesity, sedentary lifestyles, high-calorie diets, and the aging of the population [8], and there is strong evidence that type 2 diabetes can be prevented through simple lifestyle changes [9, 10]. This evidence also provides a basis for identifying high-risk individuals for whom to implement early lifestyle interventions in order to prevent type 2 diabetes altogether.

Risk prediction models may also be able to contribute to the decision-making process involved in the clinical management of patients. Health care interventions or lifestyle changes can target those who are at increased risk for type 2 diabetes or for poor prognoses. Although a variety of early prediction models have already been constructed for type 2 diabetes [11–13], they have yet to be applied in clinical practice due to their lack of predictive accuracy and successful data validation. Furthermore, the predictive value of various risk prediction models may not carry over from one population to another. At present, the amount of related research in China is limited, and there is no research on eastern China specifically.

Therefore, this study takes a new approach and constructs a precise personalized type 2 diabetes predictive model based on repeated medical examination data from residents of eastern, which can be used to guide early lifestyle intervention that can enable predictive, preventive, and personalized medicine in future.

## Methods

### Data

The physical examination records used in this study come from the database of the Health Management Center, Drum Tower Hospital Affiliated to Nanjing University Medical School, Nanjing, Jiangsu, China. The data include the physical examination records of 60,740 individuals from 2006 to 2020. Participants provided written informed consent to use their data for the study, and this research protocol was approved by the Nanjing Drum Tower Hospital Institutional Review Board.

### Data preprocessing

This study first selected individuals who had repeated physical examination data for 2 years or more. According to the information that most individuals had in their records, this study selected 31 items to include in the study: gender, age, BMI, systolic blood pressure (SBP), diastolic blood pressure (DBP), alanine transaminase (ALT), creatinine (CREA), triglycerides (TG), cholesterol (CHOL), HDL, LDL, glucose (GLU), hemoglobin A1c (HbA1c), lymphocyte (LY), granulocyte (GR), percentage of granulocyte (GR%), monocytes (MO), percentage of monocytes (MO%), eosinophil (EOS), percentage of eosinophil (EOS%), basophil (BA), percentage of basophil (BA%), mean corpuscular hemoglobin (MCH), HB, hematocrit (HCT), mean corpuscular volume (MCV), mean corpuscular hemoglobin concentration (MCHC), red blood cell volume distribution width (RDW), blood platelets (PLT), red blood cell (RBC), and white blood cell count (WBC). Individuals with incomplete data on these items were excluded from the study. After these exclusions, there were 15,166 nondiabetic patients. During the follow-up period of 1 to 14 years, 623 (4.1%) individuals developed type 2 diabetes, which defined as a fasting blood glucose level ≥ 7.0 mmol/l, glycosylated hemoglobin ≥ 6.5% or self-reported type 2 diabetes. According to whether the last physical examination showed diabetes, data from the first physical examination data of all individuals were divided into a nondiabetic group (no diabetes) and a new-onset diabetes (new diabetes) group.

### Statistical analysis

In this study, RStudio (https://www.rstudio.com) was used to carry out the statistical analysis. The characteristics of all participants were expressed as the mean (SD) for continuous variables and the frequency (percentage) for categorical variables. One-way analysis of variance and Kruskal–Wallis test were used to analyze the difference between the continuous variables with normal distributions and skewed distributions, and the chi-square test was performed to help analyze categorical variables (Table 1).

**Table 1:** Baseline characteristic according to the incidence of type 2 diabetes over 14 years (*N* = 15,166)

Characteristic	No diabetes	New diabetes	*P* value
Number	14,543	623	
Gender (Female/Male)	5526/9017	143/480	0.000
Age (year)	46(14.231)	57 (13.154)	0.000
BMI (kg/${\mathrm{m}}^{2}$)	23.9(3.167)	26.0(3.413)	0.000
SBP (mmHg)	123(17.538)	136(19.133)	0.000
DBP (mmHg)	78(11.513)	84(11.892)	0.000
ALT (U/L)	24.19(22.736)	31.29(24.754)	0.000
CREA (umol/L)	67.17(15.855)	70.66(14.273)	0.000
TG (mmol/L)	1.41(1.089)	1.93(1.477)	0.000
CHOL (mmol/L)	4.71(0.876)	4.78(0.959)	0.019
HDL (mmol/L)	1.31(0.354)	1.16(0.307)	0.000
LDL (mmol/L)	2.61(0.697)	2.65(0.756)	0.201
GLU (mmol/L)	5.07(0.544)	5.98(0.616)	0.000
HbA1c (%)	5.56(0.337)	6.03(0.322)	0.000
LY (10^9/L)	2.09(0.596)	2.22(0.670)	0.000
GR (10^9/L)	3.53(1.113)	3.85(1.275)	0.000
MO% (%)	5.73(2.072)	5.72(2.088)	0.859
EOS% (%)	2.25(1.868)	2.32(1.937)	0.251
BA% (%)	0.36(0.262)	0.38(0.264)	0.022
MO (10^9/L)	0.38(0.130)	0.41(0.131)	0.000
MCH (pg)	30.25(1.888)	30.54(1.734)	0.000
HB (g/L)	145.81(15.017)	149.77(14.058)	0.000
HCT (%)	38.07(14.987)	37.52(16.937)	0.346
MCV (fl)	90.15(7.602)	90.94(4.618)	0.002
MCHC (g/L)	335.65(12.239)	335.84(11.772)	0.646
RDW (%)	12.40(2.638)	12.36(3.009)	0.673
PLT (10^9/L)	221.55(53.212)	214.73(57.565)	0.001
BA (10^9/L)	0.014(0.022)	0.019(0.026)	0.000
EOS (10^9/L)	0.15(0.128)	0.17(0.174)	0.000
GR% (%)	53.55(15.025)	53.31(16.344)	0.675
RBC (10^12/L)	4.83(0.475)	4.91(0.465)	0.000
WBC (10^9/L)	6.18(1.482)	6.68(1.707)	0.000

Data are shown as means (SD), *P* value

*SBP*, systolic blood pressure; *DBP*, diastolic blood pressure; *ALT*, alanine transaminase; *CREA*, creatinine; *TG*, triglyceride; *CHOL*, cholesterol; *GLU*, glucose; *HbA1c*, hemoglobin A1c; *LY*, lymphocyte; *GR*, granulocyte; *MO*, monocytes; *EOS*, eosimophil; *BA*, basophil; *MO*, monocytes, *MCH*, mean corpuscular hemoglobin; *HCT*, hematocrit; *MCV*, mean corpuscular volume; *MCHC*, mean corpuscular hemoglobin concentration; *RDW*, red blood cell volume distribution width; *PLT*, blood platelet; *RBC*, red blood cell; *WBC*, white blood cell count

Additionally, the study further used least absolute shrinkage and selection operator (LASSO) regression to reduce high-dimensional data, and selected features with non-zero coefficients in the LASSO regression model as the most useful candidate predictors for type 2 diabetes. The study then combined the candidate predictors with multivariate logistic regression analysis, and the OR (95% CI) and *P* value of each candidate predictor variable were calculated in order to predict each patient’s possible diagnosis. Finally, the study established a nomogram of the type 2 diabetes prediction model based on these predictor variables.

Furthermore, this study evaluated the performance differentiation, calibration, and clinical validity of the nomogram. First, a calibration curve was plotted to evaluate the calibration ability of the type 2 diabetes nomogram prediction model. The area under the ROC curve (AUC) using 500 bootstrap resampling was plotted in order to quantify its discriminative performance. Second, a decision curve was drawn to evaluate the nomogram’s clinical validity. Subtracting the proportion of false-positive results from the proportion of true positive results, and then, weighing the relative risks of false-positive and false-negative results, the net benefit of each decision was obtained.

## Results

### Baseline characteristics

From 2006 to 2020, 15,166 individuals who went to the health management center of the hospital for physical examination were nondiabetic individuals at the first physical examination. Among them, 623 (4.1%) individuals were diagnosed with type 2 diabetes at some point during the next 1 to 14 years. According to whether the last physical examination had diabetes, the first physical examination data of all individuals was divided into the nondiabetic group (no diabetes) and the new-onset diabetes (new diabetes) group. Baseline characteristics of the study individuals in the two groups are reported in Table 1.

### Character selection and development of an individualized prediction model

**Fig. 1:**
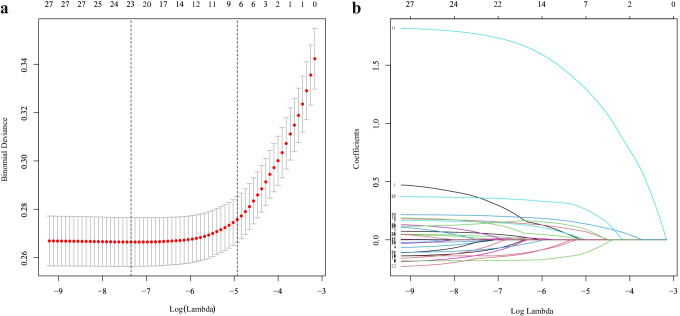
Feature selection using a LASSO binary logistic regression model. **a** The optimal parameter (lambda) in the LASSO model was selected by five-fold cross-validation. Plot binomial deviation versus log (lambda). A dashed vertical line is drawn at the optimal value by using the smallest criterion (left dashed line) and one standard error of the smallest criterion (1-SE criterion) (right dashed line). The minimum criterion refers to one of all lambda values to obtain the mean of the minimum target parameter. The 1-SE criterion refers to the lambda value of the simplest model within the minimum criterion variance. **b** LASSO coefficient curve for 31 features. Coefficient distribution plots were generated for the log(lambda) series. Plot vertical lines at the values chosen using five-fold cross-validation where the best lambda results in 10 features with non-zero coefficients for building the predictive model

**Fig. 2:**
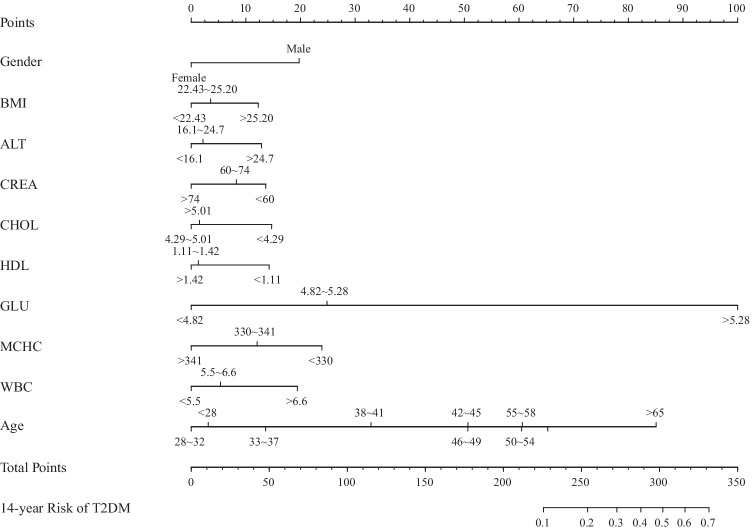
Nomogram for predicting 14-year risk of type 2 diabetes in non-diabetic individuals. To estimate an individual’s 14-year risk of type 2 diabetes, first find the corresponding value on each variable axis of the nomogram, and then draw a vertical line upward to get the corresponding points, and find the corresponding points for each variable. Finally, adding the points of all variables gives the total points of the individual, and based on the total points, a vertical line is drawn downward to obtain the 14-year risk of type 2 diabetes for the individual

For the recorded characteristics of population statistics, diseases, and therapeutic features, the study effectively reduced 31 features to 10 potential predictors (Fig. 1a and b) that have non-zero coefficients in the Lasso regression model. The 10 potential predictors were gender, BMI, ALT, CREA, CHOL, HDL, Glu, MCHC, WBC, and age (Table 2). A nomogram for predicting the 14-year risk of type 2 diabetes was developed based on these 10 independent predictors, as shown in Fig. 2.

**Table 2:** Multivariate logistic regression analysis of risk factors associated with type 2 diabetes over 14 years

	*β*	Odds ratio(95% CI)	*P* value
Gender	0.550	1.732(1.339–2.242)	0.000
BMI	0.191	1.210(1.066–1.374)	0.003
ALT	0.200	1.222(1.084–1.377)	0.001
CREA	− 0.194	0.824(0.716–0.918)	0.007
CHOL	− 0.193	0.824(0.740–0.918)	0.000
HDL	− 0.233	0.792(0.702–0.894)	0.000
GLU	1.787	5.971(4.915–7.254)	0.000
MCHC	− 0.339	0.712(0.634–0.801)	0.000
WBC	0.283	1.327(1.192–1.476)	0.000
Age	0.236	1.266(1.216–1.317)	0.000

Data are shown as *β*, odds ratio (95% CI), *P* value

*ALT*, alanine transaminase; *CREA*, creatinine; *CHOL*, cholesterol; *GLU*, glucose; *MCHC*, mean corpuscular hemoglobin concentration; *WBC*, white blood cell count

### The performance of the nomogram in the cohort study

**Fig. 3:**
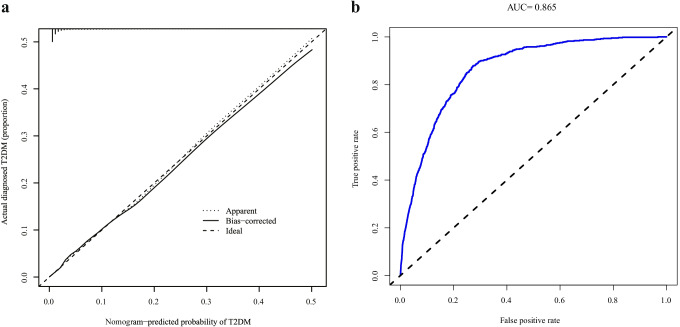
Calibration and ROC curves of the nomogram for 14-year type 2 diabetes risk. **a** Calibration curves of the nomogram for 14-year type 2 diabetes risk. The *x*-axis represents the predicted 14-year risk of type 2 diabetes. The *y*-axis represents the actual diagnosed type 2 diabetes. Diagonal dashed lines represent perfect predictions from the ideal model. The solid line represents the performance of the nomogram, where the dashed line closer to the diagonal represents better prediction. **b** ROC curves of the nomogram for 14-year type 2 diabetes risk. The AUC of the nomogram is 0.865 (95% CI: 0.847–0.883) using bootstrap resampling (times = 500). ROC: receiver operating characteristics curves; AUC: area under curve

The calibration curve for predicting the risk line diagram of the risk line chart of future type 2 diabetes patients showed good consistency in the queue (Fig. 3a). Using an internally validated bootstrap sampling method, we found the AUC of the nomogram to be 0.865 (95% CI: 0.847–0.883), which indicates that the model has good predictive capabilities (Fig. 3b).

### Decision curves for the nomogram

**Fig. 4:**
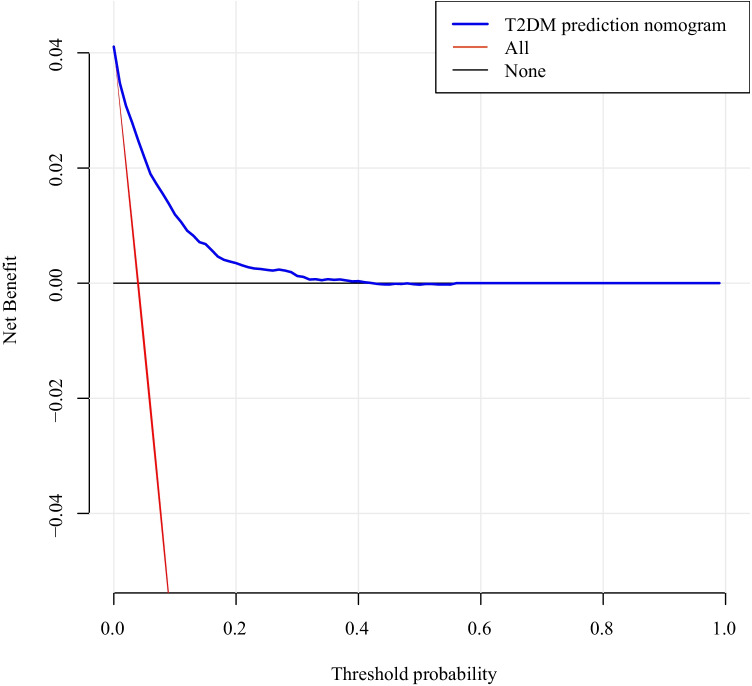
Decision curve analysis of the nomogram for 14-year type 2 diabetes risk. The horizontal line indicates that all participants were considered free of type 2 diabetes, and when no intervention was performed, the net benefit was 0. The slashes represent the net benefit when all participants were considered to have type 2 diabetes and all received the intervention. The further the model curve is from these two lines, the better the clinical value of the nomogram

Decision curve analysis (DCA) of the 14-year type 2 diabetes risk prediction nomogram model is shown in Fig. 4. The abscissa is the threshold probability, and the ordinate is the net profit rate after subtracting the pros and cons. The two straight lines in the figure represent the two extreme cases. The horizontal line indicates that all participants being considered free of type 2 diabetes, and thus the net benefit is 0 when no intervention is performed. The slashes indicate the net benefit when all participants are considered to have type 2 diabetes and all received the intervention. The model curve is thus compared to these two lines. The farther the model curve is from these two lines, the better the clinical benefit of the nomogram. For example, at a risk threshold of 10%, the net benefit is about 2%.

## Discussion

PPPM is a holistic strategy in healthcare that aims to predict individual susceptibility, provide targeted prevention, and create personalized treatments [14]. T2DM is usually treated after disease onset, which is a very delayed approach from a PPPM perspective [15]. In an effort to remedy this, in this study, we developed a simple, quantifiable, and clinically beneficial nomogram to predict the 14-year risk of T2DM in nondiabetic populations of eastern China. Both the calibration curve and AUC values indicate that the nomogram had good calibration and has good predictive capabilities. In addition, the DCA shows that the nomogram has good clinical application value. The predictive model can help to predict the incidence of T2DM, and early intervention may help at-risk individuals avoid the occurrence of T2DM-related complications.

The prevalence of T2DM continues to rise [1], and effective prevention of T2DM is essential to reduce the overall impact of this disease. Identifying high-risk groups through risk prediction methods and intervening early can help reduce the psychological stress experienced by patients, can help enhance their confidence in following a healthy lifestyle, and can help improve their quality of life. Such intervention can also delay disease progression and reduce the risk of life-long complications. In the past few decades, various predictive models have been developed to predict the occurrence of T2DM. Well-known examples include the Finnish Diabetes Risk Score [16], Australian Type 2 Diabetes Risk [17], QRISK[18], and Framingham Offspring (FOS) Risk [19]. Most T2DM prediction models use logistic [20–22] or Cox regression [23–25] and use carriage return and automatic direction forward selection, backward elimination, or step-by-step procedures.

In this study, the nomogram first constructs a multi-factor regression model (LASSO regression and logistic regression), and assigns each value level of each influencing factor according to the degree of contribution of each influencing factor in the model to the outcome variable (the size of the regression coefficient). It then assigns a score to each value level of each influencing factor and sums these scores to obtain a total score. Finally, the predicted value of the individual outcome event is calculated through the function conversion relationship between the total score and the probability of the outcome event. Simply put, the nomogram transforms complex regression equations into a visual graph, making the results of the predictive model more readable and possibly better facilitating patient evaluation. The intuitive and easy-to-understand characteristics of the nomogram have gradually garnered it more and more attention and application in both medical research and clinical practice [26, 27]. In addition, all previous T2DM risk prediction studies conducted in China have established T2DM risk scores of integer points or segment values, and the nomograms can provide more accurate and personalized risk predictions due to the use of continuous values. This is in line with EPMA. The same point of view is that individualization should become a general social trend in the field of medicine and healthcare [28].

At present, nomogram has been previously studied in the field of T2DM in China [29–31], but existing nomograms cannot be extended from one region to another. In particular, there has never been a nomogram study in eastern China. Compared to existing nomogram studies for other regions, this study has the highest AUC value and the longest follow-up time (14 years) used so far. Furthermore, risk factors included in this nomogram were gender, BMI, ALT, CREA, CHOL, HDL, Glu, MCHC, WBC, and age, which provide greater detail than the existing studies. Among them, gender, BMI, HDL, GLU, and age have been also included in other predictive models [29–31]. In addition, the studies have also shown that impaired liver function is associated with T2DM [32]. And CREA [33], WBC [34], and MCHC [35] are also related to T2DM. Therefore, the application of these parameters in the model appears to be well-founded. Finally, the calibration curve shows that the nomogram is well-calibrated, and the AUC shows its statistical accuracy. However, accuracy does not necessarily mean that it has value in clinical applications. For this reason, we also conducted a decision curve analysis, and this showed that the nomogram indeed has good clinical utility.

Although this model’s predictive results are good, a key limitation of this study is that the risk of T2DM was predicted based only on laboratory data, and did not include factors such as diet, exercise, or genetics that have been shown to be closely related to T2DM because these data were not collected. High-risk groups are typically in an intermediate physical state between ideal health and disease, also known as suboptimal health status (SHS). As a subclinical stage of chronic disease, from the perspective of PPPM, early identification of SHS is of great significance for targeted prevention and individualized treatment of T2DM [36]. The most widely used SHS screening tool is the Suboptimal Health Status Questionnaire 25 (SHSQ-25), but we also did not collect responses to this questionnaire in this study. Another limitation is that the diagnosis of T2DM could only rely on fasting blood glucose, glycosylated hemoglobin, and medical history from physical examination data because the physical examination population could not perform the standard OGTT test for the diagnosis of T2DM. In addition, this study did not divide into two groups for internal verification and external verification.

Diabetes is a metabolic disease caused by the interaction of genetic and environmental factors [37], and epigenetic modifications have been shown to be associated with its pathogenesis [38]. Epigenetics affects life activities through DNA methylation, histone acetylation, and methylation modification, and its changes also affect the occurrence and development of diabetes. In addition, environmental factors can significantly increase the risk of T2DM by affecting DNA methylation and histone modification [39]. In view of the fact that the metabolic processes and interactions of life activities and healthy cells, including the occurrence of various human diseases, depend on changes in the molecular structure of polysaccharides, glycomics (the study of the molecular structure of polysaccharides) is used in the field of biomedicine. Genetic and other factors influence glycosylation and, in turn, whether glycoproteins trigger anti- or pro-inflammatory responses [40]. This is associated with various diseases such as biological aging, metabolic syndrome, and coronavirus disease 2019 (COVID-19) [41–44]. Immunoglobulin G fragment crystallizable (IgG Fc) glycosylation has potential as a biomarker for T2DM [40], and the integration of glycomics with other biomarkers may offer further hope for future T2DM PPPM. In the future, our group intends to adopt a comprehensive glycomics strategy to study the changes in the blood glycosylation of T2DM patients, which may help us to understand the complex physiological changes of T2DM and provide better PPPM.

## Expert recommendations

As suggested in the “PPPM in Diabetes Mellitus” EPMA white paper from 2012 [7], treatment measures focused on prevention and early identification methods deserve due consideration, and a central component of prevention strategies is identification of individuals at risk for diabetes mellitus. This study constructed a T2DM prediction model in line with the recommendations in the “patient-specific modeling” paper of the EPMA that focused on research and development for the predictive and preventive potential of new IT tools for in vitro and in vivo diagnostics and the consequent evaluation and implementation of these tools in daily healthcare. The model constructed in this study can predict individuals at high risk of T2DM, and personalized medical guidance for these high-risk individuals can be furnished to primary can providers to help prevent T2DM. In subsequent work, we aim to include other data (such as OGTT and eating habits) in the model with the goal of further optimizing it.

## Conclusion

In conclusion, this study is based on a relatively large sample size and the longest yet reported follow-up time of 14 years to predict the risk factors for T2DM. We developed a nomogram prediction model for the occurrence of T2DM in the nondiabetic population of eastern China within this 14-year sample period. This nomogram may therefore be able to be used as a simple, economical, and widely scalable tool to predict personalized 14-year risk of T2DM in eastern China, thereby providing strategies for treatment from a PPPM perspective.

## Data Availability

All requests for data are made upon request, and access to relevant data can be requested through the corresponding author.
