# Correction to: A nomogram model for the risk prediction of type 2 diabetes in healthy eastern China residents: a 14‑year retrospective cohort study from 15,166 participants

**DOI:** 10.1007/s13167-022-00297-y

**Published:** 2022-08-24

**Authors:** Tiancheng Xu, Decai Yu, Weihong Zhou, Lei Yu

**Affiliations:** 1 Department of Hepatobiliary Surgery, Nanjing Drum Tower Hospital, The Affiliated Hospital of Nanjing University Medical School, No. 321 Zhongshan Road, Nanjing, China; 2 Department of Health Management Centre, Nanjing Drum Tower Hospital, The Affiliated Hospital of Nanjing University Medical School, No. 321 Zhongshan Road, Nanjing, China

**Correction to: EPMA Journal (2022)**

**https://doi.org/10.1007/s13167-022-00295-0**

In this article the author name ‘Tiancheng Xu’ was incorrectly written as ‘Tianchen Xu’.

The original article has been corrected.

